# Decoding the Apical–Basal Surfaceome of Colon Epithelial Cells via Side-Selective Biotinylation

**DOI:** 10.3390/biom16060865

**Published:** 2026-06-12

**Authors:** Katalin Kuffa, Tamás Langó, András Czirók, Júlia Tárnoki-Zách, Szilvia Bősze, Loretta László, Virág Vas, Zoltán Szabó, Gábor E. Tusnády

**Affiliations:** 1 Doctoral School of Biology, Institute of Biology, ELTE Eötvös Loránd University, Pázmány P. stny. 1/C, H-1117 Budapest, Hungary; 2 Institute of Molecular Life Sciences, Research Centre for Natural Sciences, HUN-REN, Magyar Tudósok krt 2, H-1117 Budapest, Hungary; 3 Department of Biological Physics, Eötvös Loránd University, Pázmány P. stny. 1/A, H-1117 Budapest, Hungary; 4 HUN-REN-ELTE Research Group of Peptide Chemistry, Hungarian Research Network, Eötvös Loránd University, Pázmány P. stny. 1/A, H-1117 Budapest, Hungary; 5 Department of Genetics, Cell- and Immunobiology, Faculty of Science, Semmelweis University, Nagyvárad tér 4, H-1089 Budapest, Hungary; 6 Department of Medical Chemistry, Albert Szent-Györgyi Medical School, University of Szeged, H-6725 Szeged, Hungary; 7 Department of Bioinformatics, Semmelweis University, Tűzoltó u. 7, H-1094 Budapest, Hungary

**Keywords:** colorectal cancer, cell surface proteins, cell polarity, apical–basolateral sides, tight junction, biotinylation, *N*-glycosylation, quantitative mass spectrometry

## Abstract

Colorectal cancer (CRC) is the third most common malignancy worldwide. Detailed characterization of cell surface proteins (CSPs) is essential for the identification of prognostic biomarkers and the development of novel therapeutic strategies. Cancer progression and epithelial cell polarity influence the expression levels and subcellular localization of these proteins. However, quantitative information on the distribution of CSPs between the apical and basolateral membranes remains limited, particularly in CRC cells. Here, we developed a rapid, high-throughput method based on the enrichment of biotinylated peptides and proteins from the apical and basolateral surfaces of polarized CRC epithelial cells (HT29 and HCT116), followed by LC-MS/MS analysis. This approach enables the simultaneous identification of the side-specific distribution of ~1200 CSPs. In addition, almost 500 potential *N*-glycosylation sites with the canonical consensus sequence of these proteins were identified, which may serve as targets for future site-specific glycosylation analyses. To evaluate the sensitivity of the method, we altered the surface proteome by generating TKS4-knockout cells and identified several surface markers whose expression levels differed significantly from those of wild-type cells. Overall, our findings provide new insights into the role of CSPs in CRC cells and gene-edited models, particularly in the context of TKS4-dependent epithelial-to-mesenchymal transition (EMT)-like phenotypes that model cancer metastasis.

## 1. Introduction

Cancer is one of the leading causes of death worldwide, and colorectal cancer (CRC) represents one of the most common malignancies, being the second leading cause of cancer-related mortality [1]. The high lethality of CRC is expected to persist in the coming decades. Early-stage CRC can often be effectively treated by surgery combined with adjuvant therapies such as chemotherapy or radiotherapy. However, recurrence is common, and the development of drug resistance significantly increases the risk of treatment failure [2]. Unfortunately, CRC is frequently diagnosed at advanced stages, when metastatic dissemination has already occurred. Tumor cells can spread through the lymphatic circulation to lymph nodes and later metastasize to distant organs, particularly the lungs and liver [3]. Moreover, CRC treatment remains challenging because currently available therapies often show limited efficacy. Therefore, identifying novel CRC-specific target proteins is essential for the development of alternative therapeutic strategies.

In CRC, many diagnostic and therapeutic approaches target cell surface proteins (CSPs) because these molecules are readily accessible to therapeutic agents. Examples include MUC1-specific antibody therapies [4], antibody–drug conjugates, such as anti-EGFR antibodies linked to aminobisphosphonates [5], and immune checkpoint inhibitors [6]. Although CAR-T cell therapies have demonstrated remarkable success in the treatment of hematological malignancies, their application in solid tumors—including lung and colorectal cancers—remains limited [7]. This limitation is primarily due to the scarcity of tumor-specific antigens. In many cases, CAR-T target antigens are also expressed in healthy tissues, leading to on-target, off-tumor toxicities [8]. Furthermore, limited antigen accessibility within solid tumors can impair CAR-T cell activation, expansion, and effector function [9]. Therefore, the identification of novel CSP targets and accessible extracellular regions is critical for the rational design of CAR-T cell therapies. Indeed, from a global pharmacological perspective, more than half of currently available drugs act on CSPs [10,11,12,13]. Consequently, understanding the molecular composition, membrane localization, and topological organization of CSPs is essential not only for basic cell biological studies, but also for the development of targeted therapeutic strategies.

CSPs include membrane-anchoring proteins (MAPs), monotopic membrane proteins (MMPs), and transmembrane proteins (TMPs), which play essential roles in mediating interactions between cells and their extracellular environment [14,15]. Their function is particularly important in biological barrier systems, which separate organs and compartments in multicellular organisms [16,17]. Epithelial cells represent the most prevalent barrier-forming cell type. In polarized epithelial cells, the plasma membrane is divided into two distinct domains: the apical membrane, which faces the external environment or internal luminal cavities, and the basolateral membrane, which interacts with neighboring cells and underlying tissues [18]. These domains are separated by tight junctions, which contain intercellular adhesion complexes such as occludin and claudins—major transmembrane proteins in this region [19,20]. Tight junctions help maintain the polarized distribution of CSPs and membrane lipids through sophisticated intracellular sorting mechanisms [21].

In cancer, however, these polarity-dependent distribution patterns can be significantly altered. For example, MUC1, a transmembrane glycoprotein normally localized to the apical surface of glandular epithelia (including bladder, breast, stomach, pancreas, ovary, and respiratory tract), becomes mislocalized and can be shed into circulation during tumor progression due to the loss of cell polarity and disruption of tissue architecture [22,23]. Therefore, precise qualitative and quantitative characterization of CSP distribution between apical and basolateral membranes is essential for understanding how epithelial barriers regulate transcellular transport [24], drug uptake and efflux processes [25], cancer progression and metastasis.

Despite the well-established biological importance of CSPs in polarized cells, their domain-specific distribution remains insufficiently characterized in the literature, particularly in CRC epithelial cells. One major challenge is the low abundance of CSPs relative to intracellular proteins [26,27], as well as their unique physicochemical properties, especially in the case of transmembrane proteins [28]. Consequently, several experimental approaches have been developed to investigate CSP expression and localization. For example, CSPs in CRC cells are frequently analyzed using flow cytometry [29,30]. Alternatively, side-selective protein tagging combined with Western blot analysis has been used to identify domain-specific surface proteins, such as PODXL and CEACAM1 on the apical membrane and EPHB4 on the basolateral membrane in Madin-Darby canine kidney (MDCKII) cells [31]. However, these methods have several limitations, including reliance on the availability of high-quality antibodies and accessible epitopes [32], significant time requirements, and often complex data interpretation.

To address these limitations, bottom-up proteomics approaches have emerged as powerful strategies for the simultaneous identification and quantification of large numbers of proteins in complex biological samples [33,34]. Spatial proteomic characterization of epithelial cells relies heavily on carefully designed sample preparation procedures. Examples include trichloroacetic acid precipitation of secreted proteins following viral infection [35], selective isolation of plasma or apical membranes [36,37], domain-selective surface biotinylation combined with SILAC labeling [31], and APEX2-based proximity labeling combined with TMT-based quantification [38]. Although isotope-labeling strategies are valuable for quantitative proteomics, they are relatively expensive and rely on isotopically labeled reagents that are often available only in limited quantities. In contrast, label-free quantification (LFQ) offers a cost-effective alternative and avoids labor-intensive workflows [39,40]. Therefore, the analytical pipeline presented in this study is based on LFQ.

In CSP studies, residue-specific biotinylation is frequently used to label extracellular protein regions. The labeled proteins or peptides are subsequently enriched via avidin–biotin affinity chromatography, and their relative abundances are quantified using LC–MS/MS combined with LFQ [28,41,42,43]. More recently, selective labeling of apical and basolateral surfaces in MDCKII epithelial cells has been investigated using Sulfo-NHS-SS-biotin [31,44,45]. These approaches are often combined with time-consuming gel-based separation methods for protein enrichment, which introduce several disadvantages. For example, abundant cytosolic proteins may remain associated with labeled membrane proteins and can be difficult to remove completely, even after extensive washing [46]. Moreover, enrichment at the protein level may limit the identification of site-specific modifications within individual proteins. A similar phenomenon has been observed in ubiquitination studies, where enrichment of ubiquitinated proteins resulted in lower detection of di-glycine-modified peptides compared with enrichment performed at the peptide level [47].

To overcome these challenges, the present study combines Sulfo-NHS-SS-biotin labeling with a gel-free sample preparation workflow and bioinformatics-based quality control, ensuring that labeling occurs specifically at the cell surface rather than within the cytosol. Both side-selectively biotinylated peptides and proteins were enriched and analyzed using LFQ-based tandem mass spectrometry. This approach was initially optimized in MDCKII cells, as previously described [45], and is extended here to CRC cell lines. Although Caco-2 cells represent a well-established and highly differentiated intestinal epithelial model that would constitute a convenient choice for cell surface proteomics studies, their proteome and membrane-associated protein composition have already been extensively characterized in previous studies [48]. Therefore, to generate novel and biologically informative data reflecting distinct colorectal cancer phenotypes, we selected the HT29 and HCT116 cell lines. HT29 cells have been demonstrated to retain the capacity for intestinal differentiation and may acquire polarized epithelial features with enterocyte-like or goblet-cell-like properties under appropriate culture conditions, including mucin production and epithelial polarization [49,50]. Conversely, HCT116 cells exemplify a more aggressive MSI-high, KRAS-mutant CRC model frequently employed in studies of invasion, tumor progression, and therapy resistance [51,52]. It is reasonable to hypothesize that these biological differences will have a significant impact on epithelial polarity and the organization of apical–basolateral membrane-associated protein networks, providing important context for comparative surfaceome analyses.

To further evaluate the sensitivity of our method to altered cellular states, we also analyzed a TKS4 knockout CRC cell line (HCT116). TKS4 is a scaffold protein containing a phox homology (PX) domain and four Src homology 3 (SH3) domains. Inactivating mutations in TKS4 cause Frank–ter Haar syndrome [53], and the protein also plays an important role in melanoma progression by promoting the formation of functional invadopodia [54]. TKS4 localizes to invadopodia—actin-rich membrane protrusions that mediate protease-dependent invasion [55]. In CRC research, HCT116 cells with CRISPR/Cas9-mediated knockout of TKS4 have previously been generated [56]. These cells exhibit significant changes in morphology, motility, and adhesion, consistent with an epithelial-to-mesenchymal transition (EMT)-like phenotype. In addition, TKS4-interacting proteins have recently been suggested as potential biomarkers for the diagnosis and prognosis of colon adenocarcinoma [57]. However, potential direct and indirect effects of TKS4 loss or gain on CSP composition have not yet been investigated.

In this study, we present a systematic approach for the qualitative and quantitative characterization of CSPs on the apical and basolateral membranes of CRC epithelial monolayers, including HT29 and HCT116 cells as well as a gene-edited HCT116 TKS4-knockout line. Using domain-selective surface biotinylation followed by enrichment of labeled peptides or proteins and nanoHPLC–MS/MS analysis, we also determined biotinylation sites and potential *N*-glycosylation sites in numerous CSPs.

## 2. Materials and Methods

### 2.1. Cell Culture

The human CRC cells (HCT116 and HT29; ATCC, Manassas, VA, USA) were cultured in Roswell Park Memorial Institute medium (Gibco, RPMI 1640 Medium, GlutaMAX Supplement, HEPES, Thermo Fisher Scientific (Waltham, MA, USA)) or McCoy’s 5A medium, containing 10% Fetal Bovine Serum (FBS, Gibco, Thermo Fisher Scientific) and 50 µg/mL Penicillin–Streptomycin (Gibco, Thermo Fisher Scientific) inside a humidified incubator with 5% CO_2_ at 37 °C (Eppendorf, Hamburg, Germany, Galaxy 170R). Filtered FBS was added in all freshly prepared media using Millex-GP syringe filter unit (0.22 µm pore size polyethersulfone membrane, Merck Millipore Ltd., Burlington, MA, USA) and 50 mL syringe (Henke-Ject Luer-lock, Tuttlingen, Germany). Cell line identity was verified by Short Tandem Repeat (STR) profiling. All experiments were performed using wild-type HCT116 and HCT116-TKS4-KO cells at passage 6. The cell populations were tested for mycoplasma contamination before each labeling experiment.

For the labeling experiments, cells were cultured on 6-well transwell inserts (4.67 cm^2^ surface area, 0.4 µm pore size, VWR) for 10 days, in order to form a cell monolayer on the semipermeable polyester membrane. Transepithelial electrical resistance (TEER) measurements were used to determine the optimal starting cell number for the appropriate transwell surface area and the optimal culture time, which is required for the construction of the confluent cell monolayer (Figure S1).

### 2.2. Side-Selective Sulfo-NHS-SS-Biotin Labeling

The used medium was removed from the apical and basolateral area of the transwell plate, then cell monolayers (both the apical and basolateral chamber of the plates) were washed twice with phosphate-buffered saline (PBS: 137 mM NaCl, 2.7 mM KCl, 10 mM Na_2_HPO_4_ and 1.8 mM KH_2_PO_4_; the first wash was pH = 7.4, the second wash was pH = 8.0).

The membrane-impermeable sulfo-NHS-SS-biotin was used to label the accessible primary-amino groups on the surface of the cells. This reagent was dissolved before use in HPMI buffer (9 mM glucose, 10 mM NaHCO_3_, 119 mM NaCl, 9 mM HEPES, 5 mM KCl, 0.85 mM MgCl_2_, 0.053 mM CaCl_2_, and 5 mM Na_2_HPO_4_ × 2H_2_O, pH 8.0) to reach the ~2 mM final concentration. The apical or basolateral sides of the cells are labeled selectively with the reagent at room temperature for 10 min. Then, 25 mM Tris buffered saline (TBS, 25 mM Tris, 150 mM NaCl; pH 7.4, Thermo Fisher Scientific) was used to stop the labeling process. The biotinylated cells were washed again three times by TBS to remove the excess of the labeling agent and the small number of damaged cells before cell lysis.

### 2.3. Cell Lysis and Membrane Preparation

After the last quenching solution was removed, the cells were scraped from the surface of the transwell inserts into ice-cold hypotonic lysis buffer (20 mM Tris–HCl, 10 mM KCl, 20 mM sucrose, 10 mM iodoacetamide (IA), pH 7.4) using a cell scraper. The labeled cells were incubated in ice-cold IA-containing lysis buffer for 10 min at 4 °C.

The solutions were centrifuged at 1700× *g* for 10 min at 4 °C, and then the cell pellets were mechanically lysed using micro pestle and 26-gauge ½ inch needle with 1 mL syringe. The intact cells, cell debris, and nuclei were pelleted at 1700× *g* for 7 min at 4 °C. The mechanical lysis was repeated once on the pellet fraction to maximize the quantity of membrane preparations. The supernatant was transferred into a 10.4 mL polycarbonate tube (Beckman Coulter, Miami, FL, USA) and centrifuged at 100,000× *g* for 1 h at 4 °C using a 70.1 Ti fixed rotor (Beckman Coulter) in an L7-55 ultracentrifuge (Beckman Coulter). The supernatant was discarded and the pellet was washed once with 10 times diluted lysis buffer without iodoacetamide (pH set to 7.7 by 1.7 M Tris stock solution). Finally, the pellet was centrifuged again at 100,000× *g* for 1 h at 4 °C. The pellet was resuspended in the diluted lysis buffer and homogenized by 25 strokes with a Potter-Elvehjem PTFE pestle in a glass tube (2 mL, Sigma-Aldrich, St. Louis, MO, USA) on ice. The membrane preparations are stored at −20 °C until further use. The protein content of the preparations was measured with Lowry method [58].

### 2.4. Labeled Protein Enrichment Sample Preparation Protocol from Membrane Preparations

Side-selectively labeled membrane preparations (75–100 μg protein/biological or technical replicates) were solubilized by 0.1% (m/V) RapiGest SF in 50 mM ammonium bicarbonate (AmBic; Sigma-Aldrich), using an ultrasonic cleaning machine (Joan Lab Equipment Co., Ltd., Huzhou, China) in a cold room. The solutions were sonicated in 5 consecutive cycles for 1 min each. To support the protein solubilization efficiency of RapiGest SF, firstly the solutions were stored on ice for 30 min (vortex every 5 min for 5 s) and secondly boiled in a dry-block thermostat for 5 min at 95 °C. The solutions were cooled down, and then IA was added in 1.25 mM final concentration and incubated at 37 °C for 30 min in dark (after the alkylation, 1.25 mM 2,2′-Thiodiethanol was used avoiding the overalkylations during the following steps). The labeled proteins were enriched on High-Capacity Neutravidin agarose resin. The optimal amount of the resin was determined by dot blot that was loaded into Pierce Snap Cap Spin-Column; the labeled proteins were also loaded and incubated at room temperature for 60 min. Thereafter, the resin was washed extensively (with at least 10 times of the resin bed volume for each wash buffer) as follows: 200 µL 50 mM AmBic (4×), 200 µL 1 M NaCl (4×), 200 µL 50 mM AmBic (4×), 200 µL 100 mM NaHCO_3_ (4×) and 200 µL 60 °C pre-heated AmBic (4×), and finally 200 µL 50 mM AmBic (2×). The enriched proteins were eluted by 10 mM Dithiothreitol (DTT, Thermo Fisher Scientific) reducing agent in 50 mM AmBic buffer for 30 min at 37 °C, this step was repeated one more time, and the solutions were combined. Eluted proteins were alkylated with 25 mM IA in dark at 37 °C for additional 30 min. Next, 7× volume of pre-cooled acetone was added to the samples, and the protein pellets were left to precipitate overnight at −20 °C. Next day, the samples were centrifuged at 14,000× *g* for 10 min at 4 °C, supernatants were discarded, and the pellets were washed two times with acetone–water (85:15%, (*v*/*v*). In every washing step the samples were vortexed and sonicated (three times, each time for 1 min). The proteins were resuspended in 0.1% (m/V) RapiGest SF in 50 mM AmBic (sonicated for 5 × 1 min), and protein contents were determined [58]. Samples were then treated by PNGaseF (for 2 h at 37 °C, 250 units/sample) and digested with proteomics-grade trypsin (1:25 (*w*/*w*) enzyme:protein ratio) and incubated overnight at 37 °C. Next day the reactions were stopped by formic acid (0.75 µL ccFA/sample), and peptide mixtures were dried using a SpeedVac concentrator (Eppendorf, Hamburg, Germany). The previously optimized C18 purification [43] was made by the following steps: C18 spin cartridges were activated and equilibrated with 2 × 200 µL 50% MeOH, 2 × 200 µL 0.5% TFA + 5% ACN, and 2 × 200 µL 0.1% HFBA in water. Then, the concentrated samples were dissolved in 50 µL 0.1% HFBA in water and loaded onto the pre-washed C18 spin column (the flow-through fraction was loaded again to the column, to minimize the peptide loss). Contaminants were washed away with 2 × 100 µL 0.1% HFBA, and peptides were eluted with 2 × 50 µL 0.1% TFA + 70% ACN solution and with 50 µL 0.1% FA + 70% ACN solution. Finally, the eluted fractions were combined and dried again using a SpeedVac concentrator and stored at −20 °C until the mass spectrometry analysis.

### 2.5. Labeled Peptide Enrichment Sample Preparation Protocol from Membrane Preparations

In this method, the labeled membrane preparations were treated as described in our previous works [41,43], with slight modifications. Briefly, 75–100 μg apical or basolateral biotinylated membranes were solubilized similarly as in the protein enrichment section. Then, the membrane proteins were first digested overnight with proteomics-grade trypsin (1:25 (*w*/*w*) enzyme:protein ratio) at 37 °C. Next day, the digestion stopped by heat inactivation for 10 min at 95 °C, followed by TLCK trypsin inhibitor treatment (~50 µM final concentration) for 45 min at 37 °C. The peptide mixtures were incubated with PNGase F for 2 h at 37 °C (250 units/sample) to remove the *N*-glycans. Then the biotinylated peptides were isolated on High-Capacity Neutravidin agarose resin and the non-specific peptides were removed by the above-mentioned washing steps, and finally eluted by 10 mM DTT in two consecutive steps. The peptide mixture was dried immediately (no acetone precipitation in this protocol), C18 desalting was made similarly as described in the case of the protein enrichment protocol, and the samples were stored at −20 °C until further use.

### 2.6. Mass Spectrometry Analysis

Isolated peptide mixtures were analyzed by the data-dependent acquisition (DDA) LC-MS method described in our previous work [45]. LC-MS measurements were performed on a Waters ACQUITY UPLC M-Class LC system (Waters, Milford, MA, USA) coupled with an Orbitrap Exploris 240 mass spectrometer (Thermo Fisher Scientific, Waltham, MA, USA). Data-dependent acquisition (DDA) measurements were collected using a method with MS1 scan between 360 and 2200 Th using 60,000 resolution, while ddMS2 scans with isolation windows of 2 Th were collected at 30,000 resolution keeping a 3 s cycle time. Raw LC-MS data files were processed using Fragpipe v22.0 [59]. Human (83,676 proteins) reference proteome assuming 2 missed cleavage sites was used for protein identification. The database searches were performed assuming carbamidomethylation of Cys as fixed, 3-(carbamidomethylthio)propanoyl Lys and protein N-term, deamidation (deglycosylation) of Asn and oxidation of Met as variable modifications. Protein quantification was performed within Fragpipe (v22.0) using IonQuant (v1.10.27) with default settings and enabling match between runs. A contaminant database was created using a database search of LC-MS data collected from a labeled and enriched digest of cell medium using the same search settings and Uniprot Bovine reference proteome database (26,942 proteins). Based on analysis of cell growth medium, a contaminant database including 290 bovine proteins, porcine trypsin (TRYP_PIG) and neutravidin (AVID_CHICK) was built. Proteins identified with bovine-specific peptides were removed from further analysis.

Statistical analysis and visualization of proteomics data was performed in Perseus 1.6.15145 [60] and Instantclue v0.12.2146 [61]. Protein intensity data were log2 transformed and normalized before statistical analysis according to the method described previously [45]. Differential expression analysis was performed using Student’s *t*-test with permutation-based false discovery rate (FDR) estimation. A significance limit of FDR < 0.05 was used in each comparison. In case of polarization analysis, a minimum of 60/40 or 40/60 apical/basolateral ratio was required in addition to FDR limit, to be considered as a significant difference. (See details in the Supplementary Materials and Methods section.)

## 3. Results

### 3.1. Workflow Optimization for CRC Surface Proteome Analysis

**Figure 1:**
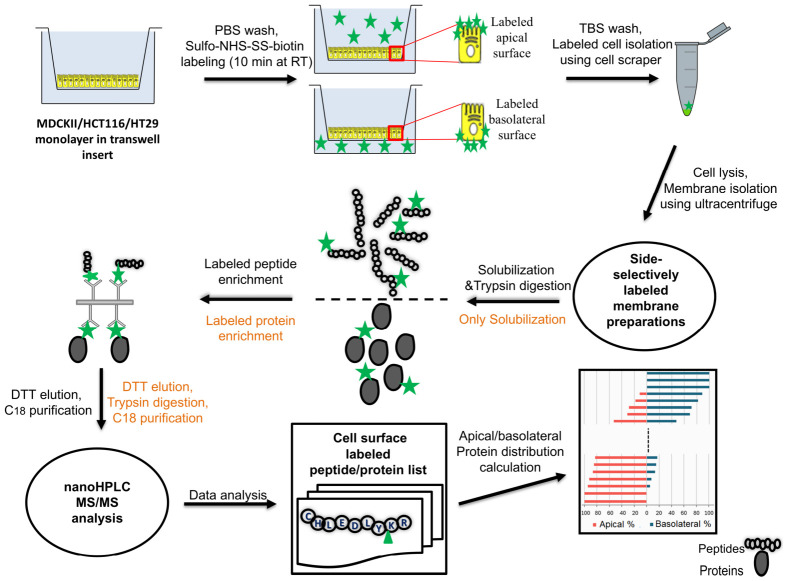
Flowchart to identify the plasma membrane-associated proteins on the apical and basolateral surface in polarized cells. Cells were labeled with a membrane-impermeable, primary amino group-specific labeling agent (Sulfo-NHS-SS-biotin, green stars) at room temperature (RT) for 10 min from apical or basolateral chamber of the transwell inserts. The reaction was stopped with TBS buffer, cells were lysed; side-selectively labeled plasma membranes were isolated. The preparations were solubilized; without digestion labeled proteins were enriched on a neutravidin agarose resin (steps are written with orange), while in the other case proteins were digested with trypsin and labeled peptides were isolated. The biotinylated peptides/proteins were eluted with DTT reducing agent (where labeled proteins were eluted, they were digested after this step), desalted on a C18 column and sequenced by nanoHPLC-MS/MS. The surface accessible protein segments and protein abundances were determined in the polarized HT29 and HCT116 cells. Based on the apical and the basolateral label-free intensity values of the proteins, the side-selective distributions of more hundreds of surface proteins were calculated. The green stars represent the Sulfo-NHS-SS-biotin labeling, and the green triangle is the remnant region of the labeling agent after reduction.

The workflow implemented for characterization of the surface proteome of CRC cells is summarized in Figure 1. Individual steps of the process had previously been optimized in our earlier studies [43,45,62]. However, when CRC cells were cultured on transwell inserts, additional optimization was required to achieve the formation of a well-sealed epithelial monolayer and reliable side-selective biotinylation.

First, TEER measurements were performed on HCT116 and HT29 cells as well as on gene-edited derivatives of HCT116 cells (Figure S1A and S1B, respectively) in order to determine the optimal culture duration required for proper monolayer formation in each cell type. Second, apical and basolateral biotinylation efficiencies were examined using a dot blot assay with HRP-conjugated streptavidin (Figure S2A). The results demonstrate that biotinylation occurred with comparable efficiency on both sides of the cell monolayer.

To minimize potential loss of labeled peptides or proteins during affinity enrichment, optimal neutravidin resin volumes were also determined using the dot blot method. These experiments demonstrated that all biotinylated components from both digested and non-digested membrane preparations were retained on the high-capacity neutravidin agarose column (Figure S2B). Both labeled protein-level and labeled peptide-level enrichment strategies were subsequently applied to all samples. The resulting C18-purified peptide mixtures were analyzed by tandem mass spectrometry to determine the qualitative and quantitative composition of the CRC cell surface proteome.

### 3.2. Protein Yields Obtained with the Developed Workflow

**Figure 2:**
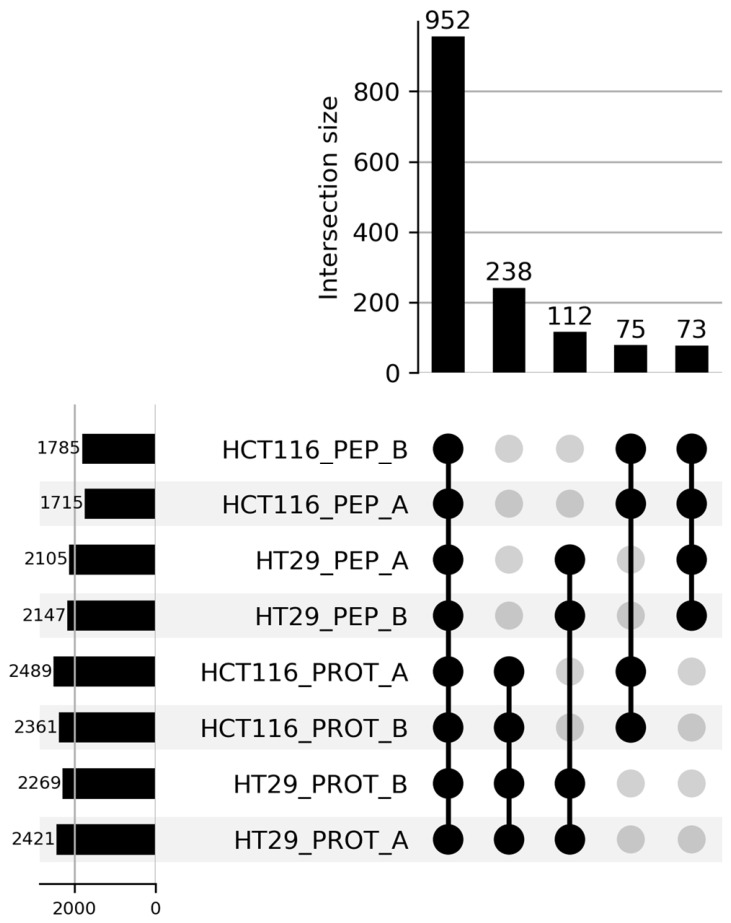
Comparison of the results of HCT116 and HT29 cell lines using biotinylated peptide-level and protein-level enrichment strategies. The figure shows the number of proteins detected from labeled peptide- and labeled protein-level enrichment strategy using HCT116 and HT29 CRC cells and the overlaps between our sample types (“PEP” means labeled peptide enrichment strategy, “PROT” means labeled protein enrichment strategy, A and B represent apically or basolaterally labeled samples).

Using the peptide-level enrichment strategy, we detected 1715 and 2105 proteins from apically labeled samples and 1785 and 2147 proteins from basolaterally labeled samples in HCT116 and HT29 cells, respectively. In contrast, protein-level enrichment yielded 2489 and 2421 proteins from the apical domains and 2361 and 2269 proteins from the basolateral domains of HCT116 and HT29 cells, respectively (including contaminating proteins). Furthermore, 527 proteins were unique to HCT116 samples and 632 proteins were unique to HT29 samples (excluding contaminants). Overall, 952 proteins out of a total of 3645 identified proteins were detected in all eight sample types (see Figure 2). Among the 952 common proteins, 424 were identified with at least one correctly localized extracellular biotinylated peptide (the correctness of extracellularly labeled positions from TMPs was checked using other experimental topology data collected in the UniTmp database [63]), indicating that these proteins are likely located on the surface of CRC cells. Across all samples, 708 proteins containing such extracellular biotinylation sites were identified (see Supplementary Table S1).

When comparing the enrichment strategies, the peptide-level enrichment method resulted in a higher number of biotinylated peptides and proteins than the protein-level enrichment approach. In total, 1551 proteins containing at least one labeled peptide were identified. Among these, 1524 were detected in peptide-enriched samples, whereas 424 were identified in protein-enriched samples.

Comparison of the two CRC cell lines revealed that more labeled proteins were detected in HT29 cells (1394 proteins) than in HCT116 cells (859 proteins) (see Supplementary Table S1).

As summarized in Table 1, the peptide-level enrichment strategy identified a larger number of biotinylated proteins and transmembrane proteins (TMPs) in both CRC cell lines. Examination of the topological correctness of the labeled peptides indicated that the majority of proteins contained at least one correctly localized extracellular labeling site, demonstrating high cell surface selectivity for most enrichment strategies (with the exception of the HT29 peptide-enrichment dataset, which is discussed in detail in the Supplementary Results).

Overall, the developed workflow enabled the analysis of hundreds of targetable cell surface proteins, many of which may play important roles in CRC development and could represent potential diagnostic or therapeutic targets.

### 3.3. Comparison with Databases Containing Polarized Cell Surface Data

To evaluate the biological relevance of our results, we compared our dataset with publicly available protein localization databases. First, we used the Compartments database [64], which integrates subcellular localization data from experimental evidence, sequence-based predictions, and automated text mining. Apical proteins were filtered based on 18 non-cytosolic apical annotations in the cellular component classification while excluding proteins that were also annotated as basolateral. Because basolateral annotations are relatively limited in this database, they were not included in this comparison. Several proteins predicted to be apically localized in the database were also detected predominantly on the apical side in our dataset (≥60% apical distribution based on average protein intensities), including epithelial membrane protein 2 (EMP2), ATP6V1B2, and CEACAM7 (Table 2). In addition, several proteins were identified as apically enriched in our dataset but were not annotated as such in the database, including well-known apical markers such as cathepsin B (CTSB), cell cycle control protein 50A (TMEM30A), and cadherin-related family member 5 (CDHR5).

Overall, more than half of the apically enriched proteins identified in our study were consistent with database annotations, while our results also provide CRC-specific polarization information for several additional proteins.

We further evaluated the cell surface specificity of our dataset using the CIRFESS database (Compiled Interactive Resource for Extracellular and Surface Studies) [65] (see Figure S3). A total of 310 proteins were detected exclusively in our CRC samples. Among these, 231 proteins had annotated subcellular localization in UniProt (cell surface, plasma membrane, extracellular space, apical or basolateral membrane, or cell junction), while an additional 43 proteins had supporting Gene Ontology cellular component annotations. These analyses indicate that the developed workflow shows high specificity for cell surface proteins while also identifying previously unreported potential CSPs in CRC cells.

### 3.4. Identification of Cell Line-Specific CSPs and Comparison of CRC Aggressiveness

In a previous study, Ludvigsen et al. [66] analyzed protein expression in a normal colon mucosa cell line (NCM460) and a primary colon adenocarcinoma cell line (SW480). They also characterized HCT116 CRC cells using LC-MS/MS after 2D-PAGE separation, identifying 901 proteins that were differentially expressed between HCT116 and NCM460 cells. Among these proteins, several membrane proteins were also detected in our dataset, and therefore we examined their presence and abundance in our samples.

In that study, 57 proteins were upregulated and 67 proteins were downregulated in HCT116 cells relative to NCM460 cells. Among the 57 upregulated proteins in [66], we detected 11 membrane proteins or CSPs in our HCT116 samples but not, or at substantially lower intensities, in HT29 samples. Among the 67 downregulated proteins, 32 were detected in our dataset, and 21 of these were detected only in HT29 cells but not in HCT116 cells (see Table 3). Importantly, the intensity values of these 23 membrane or cell surface proteins were generally higher in HT29 cells than in HCT116 cells.

These observations are consistent with the notion that HCT116 cells exhibit a more aggressive phenotype than HT29 cells, since the HT29 expression pattern appears more similar to that observed in normal colon epithelial cells.

We also identified cell line-specific CSPs. For example, 23 labeled membrane proteins containing at least one correctly localized extracellular biotinylation site were detected exclusively in HCT116 cells, including PLAU, CD40, ROR2, NECTIN3, and ICAM5. Conversely, 34 labeled membrane proteins were detected only in HT29 cells, including LY75, AGR2, CEACAM5, CD14, LGALS4, and TMPRSS4 (see Supplementary Table S1: CRC cell-specific labeled CSPs).

These proteins may therefore serve as potential cell line-specific surface markers. Many of them have previously been implicated in CRC progression, migration, and metastasis, as discussed in the Discussion Section.

### 3.5. Detected CRC Cell Surface Proteins in Comparison with Other Studies

Aberrant glycosylation of CSPs is a well-known feature of cancer. For example, glycoproteins carrying Tn (GalNAc) and sialyl-Tn (STn) antigens can be captured by macrophage galactose-type lectin (MGL). In a previous study, HCT116 and HT29 cell lysates were analyzed using MGL pull-down experiments following PNGase F treatment, and the top 20 interacting proteins were identified by mass spectrometry [67].

We examined whether these proteins were also detected in our dataset and whether their extracellular domains were accessible to labeling by the membrane-impermeable reagent Sulfo-NHS-SS-biotin (see Table 4).

Our results show that all lectin-enriched proteins identified in that study were also detected using our cell surface labeling strategy. Most proteins contained at least one extracellularly labeled peptide. Two exceptions were ATP1A1 and FLNB, which showed less reliable labeling in HT29 cells, possibly due to increased cellular sensitivity to labeling conditions or higher levels of cell damage during culture. Most of these proteins exhibited no strong polarization, with apical abundance values close to 50%, suggesting approximately equal distribution between apical and basolateral domains. However, PTK7, TFRC, and MET showed clear apical enrichment, whereas FLNB exhibited basolateral enrichment. Interestingly, previous studies have reported inconsistent polarization patterns for TFRC. Some reports detected TFRC at the apical surface using antibody staining [69,70], whereas others reported basolateral localization in kidney cells [71] and Caco-2 cells [72]. In our CRC models, TFRC showed a slight apical bias. We also identified *N*-glycosylation sites for several of these proteins, including PTK7, MET, ITGB1, PTGFRN, TFRC, LAMP1, SLC3A2, PTPRF and PTPRK (Supplementary Table S3). These glycosylation sites may represent potential therapeutic targets for lectin-based or antibody-based CRC therapies. In addition to validating proteins detected by lectin enrichment studies, our dataset also contains numerous proteins relevant to other CRC biomarker studies.

For example, a recently developed microfluidic platform enabled efficient exosome enrichment, which was validated using a specific exosomal marker CD9, and identified SORL1 protein as a potential biomarker for early CRC detection [73]. Using our method, both CD9 and SORL1 were detected on the surface of CRC cells, indicating that these proteins are not only exosomal markers but also potential cell surface therapeutic targets. Similarly, we detected MET (hepatocyte growth factor receptor) with 28 extracellularly labeled peptides across 48 samples.

Elevated HGF/MET signaling has previously been associated with poor overall and disease-free survival in CRC patients [74]. Consistent with these findings, our data showed higher MET levels in HCT116 cells than in HT29 cells, supporting the more aggressive phenotype of HCT116 cells reported in earlier invasion assays [75].

We observed similar trends for PTK7, which was approximately 4-fold higher in peptide-level enrichment and 6-fold higher in protein-level enrichment in HCT116 cells compared with HT29 cells. PTK7 overexpression has previously been associated with tumor differentiation, lymph node metastasis, distant metastasis, and higher TNM stage in CRC [76].

In contrast, CD46 levels were higher in HT29 cells. This observation is consistent with previous studies reporting that HCT116 cells exhibit relatively low CD46 expression compared with other CRC cell lines [77].

Another notable example is ALCAM (CD166), which is strongly expressed in intestinal stem cell niches and throughout CRC progression [78]. In our dataset, ALCAM exhibited more than an order-of-magnitude higher abundance in HCT116 cells, with a total of 254 extracellular peptides identified.

Similarly, we detected high numbers of extracellular peptides for ITGB1 and EPCAM, both of which are well-established CRC-associated proteins. ITGB1 has been shown to promote CRC growth and invasion via the Hedgehog signaling pathway [79], while EPCAM targeting has been explored in combination therapies for metastatic CRC [80].

We also detected EGFR, a transmembrane glycoprotein commonly overexpressed in CRC tumors [81]. EGFR overexpression occurs in approximately 60–80% of CRC cases and is associated with poor prognosis [82]. In our dataset, EGFR was identified with 100 extracellular peptides, including 47 labeled peptides.

Additionally, several potential CRC surface biomarkers reported in recent studies were also detected in our dataset, including GPRC5A, EPHB4, ANXA2, ANXA4, and PROM1 (CD133) [83].

Taken together, these results confirm the ability of the presented method to map relevant surface proteins in colorectal cancer, some of which may serve as diagnostic and therapeutic biomarkers.

### 3.6. Identification and Distribution of N-Glycosylation Sites

Aberrant glycosylation is widely recognized as a hallmark of cancer, and tumor-associated glycosylation patterns have emerged as promising targets for immunotherapy [84]. Using the developed peptide- and protein-level enrichment strategies in combination with PNGase F digestion, we were able to identify *N*-glycosylation sites in CRC cell surface glycoproteins in addition to biotinylated peptides. In total, 498 *N*-glycosylation sites containing the canonical N-X-S/T/C consensus sequence or the less common N-X-V motif were detected. Among these, 333 sites were located at topologically correct extracellular positions.

In HCT116 cells, 686 *N*-glycosylation sites were identified in 409 proteins, while in HT29 cells, 581 *N*-glycosylation sites were detected in 370 proteins. Detailed statistics regarding *N*-glycoproteins, N-glycosylation sites, and transmembrane glycoproteins are summarized in Table 5.

Comparison of the two CRC cell lines revealed cell line-specific glycoproteins, including 168 proteins unique to HCT116 cells (332 *N*-glycosylation sites) and 129 proteins unique to HT29 cells (227 *N*-glycosylation sites).

Some glycoproteins were detected on both apical and basolateral membrane domains, whereas others showed domain-specific localization.

For example, in HCT116 cells, several glycoproteins were detected exclusively in apical samples, including EFNA3, DSG2, SLC15A1, CSPG4, and MAGI1, whereas others were detected only in basolateral samples, such as ANTXR1, LRP11, and TMEM106B.

A total of 46 *N*-glycoproteins were detected in all four HCT116 sample types (apical and basolateral peptide- and protein-level enrichment). Among these proteins, several *N*-glycosylation sites were consistently detected across all six apical and six basolateral peptide-enriched samples, including: N286 in CADM4; N229 in NPTN; N68 and N419 in CD109; N107, N656, and N926 in ITGA3.

However, certain *N*-glycosylation sites showed domain-specific detection patterns. For example, the transmembrane receptor MET contains a known glycosylation site at N879, which was detected only in HCT116 apical peptide-level enriched samples. Conversely, N307 of the poliovirus receptor (PVR) was detected exclusively in basolateral peptide-enriched samples (Supplementary Table S3).

In HT29 cells, 44 glycoproteins were detected across all sample types (apical and basolateral peptide- and protein-level enrichment). Several glycosylation sites were consistently detected across all six apical and six basolateral peptide-enriched samples, including N278, N439, and N551 in ADAM10, as well as N418 and N840 in ITGA1.

### 3.7. Effects of TKS4 Knockout on Cell Surface Proteins

As previously described, TKS4-knockout HCT116 cells exhibit an EMT-like phenotype, similar to the cellular changes observed during cancer metastasis.

We validated the Tks4 absence/presence in these cells similarly as described previously [56,85] (see Figure S5).

In our results, although monolayers were formed, side-selective labeling of the TKS4-KO cells was less efficient, likely because less tight cell contacts allowed biotinylation reagents to access both membrane domains to some extent. Therefore, our analysis focused on changes in CSP abundance rather than polarization.

To investigate whether loss of TKS4 influences the composition of the cell surface proteome, we compared wild-type (WT) HCT116 cells with HCT116-TKS4-KO cells using the developed workflow. In total, 510 proteins were detected exclusively in WT cells, 148 proteins exclusively in KO cells, and 1769 proteins were detected in both cell types (see Supplementary Table S2).

**Figure 3:**
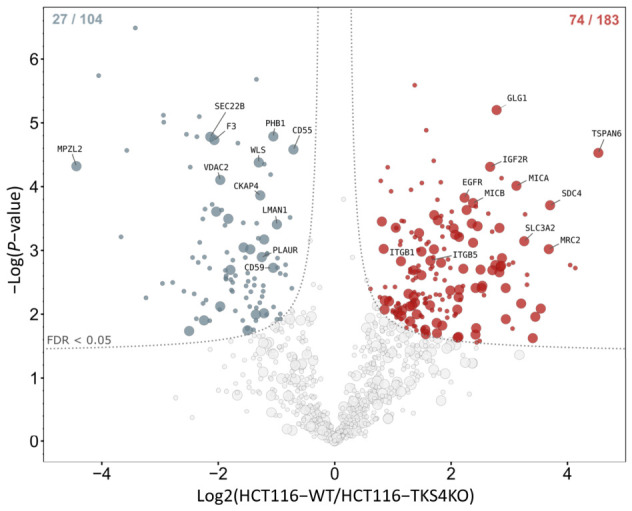
Volcano plots of protein expression changes between WT- and TKS4-KO HCT116 cells. Significance limits (FDR < 0.05) are shown by dotted lines; significantly increased or decreased proteins are colored red or blue, respectively. Biotinylated TMPs are shown with large dots. Numbers of biotinylated TMPs/proteins with significant change are shown at the top corners.

Statistical analysis identified 104 proteins significantly downregulated, and 183 proteins significantly upregulated in WT cells compared with TKS4-KO cells, including 27 and 74 transmembrane proteins with detected biotinylation, respectively (Figure 3).

## 4. Discussion

### 4.1. Method Validation and Applicability to CRC Surface Proteomics

In our previous work, we demonstrated that the cell surface protein (CSP) selectivity of the succinimide ester-based cell surface labeling strategy is satisfactory following several optimization steps [41,43,62]. In a more recent study [45], we further confirmed that, in addition to qualitative characterization (i.e., detection of extracellularly labeled peptides), this strategy also enables quantitative analysis of apical–basolateral CSP distributions. The results obtained for MDCKII cells showed strong agreement with previously published datasets, including those reported by Caceres et al. [31] and Koetemann et al. [44], as well as polarization data from the PolarProtDb database [18]. Based on these results, the method appeared suitable for high-throughput analysis of CSPs in additional epithelial cell models.

In the present study, we extended this approach to two CRC epithelial cell lines (HCT116 and HT29) and to a gene-edited derivative of the HCT116 cell line (HCT116-TKS4-KO). Both labeled peptide-level and labeled protein-level enrichment strategies were applied to obtain a more comprehensive characterization of the surface proteome of these cells.

Before labeling, we first determined the time required for the formation of confluent epithelial monolayers. TEER measurements indicated that approximately 10 days were required for stable monolayer formation in all cell types examined (Figure S1). Dot blot experiments confirmed that the apical and basolateral surfaces exhibited comparable biotinylation efficiency during the labeling procedure (Figure S2A). The same approach was used to determine the optimal amount of high capacity neutravidin agarose required for complete binding of labeled peptides or proteins (Figure S2B). Following these optimizations, we were able to quantitatively characterize thousands of proteins from CRC cell surfaces (Supplementary Tables S1 and S2). Differentially expressed proteins were also verified in the HCT116-TKS4-KO cells (Figure 3).

When comparing enrichment strategies, peptide-level enrichment consistently produced more labeled peptides per sample than protein-level enrichment, which is in agreement with our previous studies. Protein-level enrichment resulted in a larger number of non-specific proteins, including unlabeled intracellular contaminants, thereby reducing surface specificity and complicating normalization and identification of extracellular biotinylation sites. From a topological perspective, peptide-level enrichment provided significantly more data for transmembrane proteins (TMPs), although with slightly lower topological accuracy (approximately 90% for HT29 and 96% for HCT116 cells; ratio of labeled TM peptides on the correct (extracellular) side versus all labeled TM peptides, see Supplementary Table S1 and Figure S4). In contrast, protein-level enrichment produced an order of magnitude fewer labeled positions, but with nearly 100% extracellular localization accuracy.

### 4.2. Polarized CSPs in CRC Epithelial Cell Lines

Using our datasets, we identified numerous significantly polarized CSPs in HCT116 and HT29 cells (Supplementary Table S1). Based on peptide-level enrichment, 143 and 284 proteins exhibited significant polarization, while protein-level enrichment identified 14 and 253 proteins, respectively. After filtering for proteins containing at least one extracellularly labeled peptide, these numbers were reduced to 53 and 71 proteins for peptide enrichment and five and 88 proteins for protein enrichment. Overall, HT29 cells displayed a higher number of polarized surface proteins, suggesting a more preserved epithelial polarity compared with HCT116 cells.

Interestingly, we detected reduced topological accuracy in the case of HT29 cells. One possible explanation is that HT29 cells may be more sensitive to the surface labeling and washing procedures used during biotinylation, potentially resulting in partial membrane damage and unintended intracellular labeling of transmembrane proteins. In contrast, HCT116 cells appeared to better tolerate the experimental conditions, leading to improved side-specific labeling fidelity. Previous studies have also reported substantial biological and stress-response differences between HT29 and HCT116 cells, including differences in cellular stress sensitivity, apoptosis regulation, and epithelial integrity [86,87].

Several proteins showed polarization patterns consistent with previous studies. For example, CXADR and EMP2 exhibited apical localization consistent with earlier reports [88,89]. Eph receptors, which play important roles in CRC progression, are known to be enriched in the apical domain of colonic epithelium [90]. Consistent with this, EPHA1 showed strong apical enrichment in HCT116 cells. We also identified DPP4 as an apically localized protein. DPP4 inhibitors have recently been shown to suppress CRC cell growth by modulating cancer-associated pathways such as ECM–receptor interactions and PI3K–AKT signaling [91].

Among basolateral proteins, HYOU1 and ENTPD2 were particularly notable. Elevated ENTPD2 expression has been shown to promote tumor growth by suppressing CD8+ T-cell activity [92].

Interestingly, CEACAM1 showed different polarization patterns between the two CRC cell lines: basolateral localization in HCT116 cells and apical localization in HT29 cells.

Overall, these findings demonstrate that our method can effectively identify both known and previously uncharacterized polarization patterns of CRC cell surface proteins. In addition to the observed differences in epithelial polarization and the CSP alterations identified between HT29 and HCT116 cells (as listed in Table 3) we also found highly consistent results with previously reported characteristics of these CRC models. Specifically, HT29 cells exhibited elevated surface expressions of MUC13, EPCAM, CDH1, and CEACAM1, all of which are associated with the maintenance of a more differentiated phenotype. In contrast, HCT116 cells showed increased surface abundance of CD44, PROM1, and ITGB1 proteins implicated in cell migration, invasiveness, and tumor plasticity. Furthermore, elevated expressions of ALCAM, EGFR, and MET were also detected in HCT116 cells, consistent with the more aggressive molecular phenotype previously described for this CRC model. The observed surfaceome heterogeneity likely reflects the combined effects of underlying genetic and phenotypic differences between CRC models, although a direct mechanistic dissection was beyond the scope of the present study.

### 4.3. Identification of N-Glycosylation Sites on CRC Cell Surface Proteins

**Figure 4:**
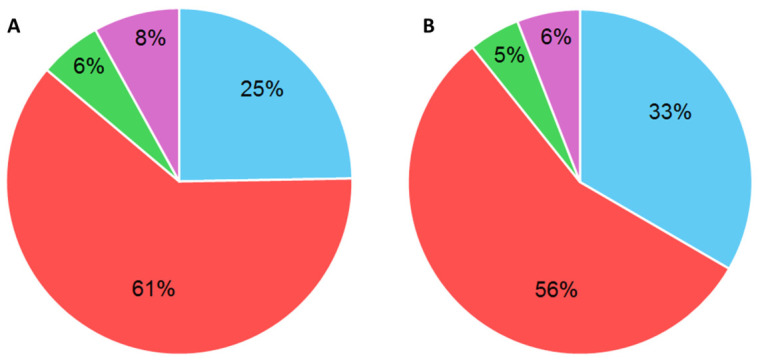
Distribution of peptides containing canonical (N-X-S/T) and non-canonical (N-X-C/V) *N*-glycosylation sites in labeled peptide enrichment samples for the two CRC cell lines. (**A**) HCT116 cells, (**B**) HT29 cells. The percentage of peptides containing deamidated asparagine originated from TMPs is shown here based on the four amino acids (T/S/C/V) at position +2 relative to the deamidated position. N-X-S/T indicated by blue and red color, respectively, N-X-C/V indicated by green and purple color, respectively.

Protein glycosylation is one of the most common post-translational modifications and plays critical roles in protein folding, adhesion, and immune recognition [93]. Aberrant glycosylation patterns are frequently associated with cancer [94]. Using PNGase F digestion combined with our enrichment strategies, we identified more than 1500 and 800 unique peptides containing deamidated asparagine residues (Supplementary Tables S1 and S2), indicating the presence of *N*-glycosylation sites. Most sites corresponded to the canonical N-X-S/T sequon, although 10–15% were in atypical motifs (Figure 4). Recent studies have demonstrated that non-canonical glycosylation sites can also be functionally relevant [95,96].

Overall, peptide-level enrichment identified 4–6 times more glycosylation sites than protein-level enrichment. Furthermore, peptide-level enrichment provided superior topological accuracy, with 98–99% extracellular localization, compared with 78–95% for protein-level enrichment.

Comparison of the two CRC cell lines revealed cell line-specific glycosylation patterns, including 95 previously unreported glycosylation sites. Notably, we identified a previously undescribed canonical *N*-glycosylation site (Asn557) in Cadherin-3, detected exclusively in HCT116 cells. Since cadherin glycoforms are emerging as potential tumor biomarkers [97], this site may represent a promising target for future CRC studies.

### 4.4. TKS4 Knockout-Dependent Surface Proteome Changes

TKS adaptor proteins are known regulators of invadopodia formation and tumor invasion [54]. Previous studies demonstrated that TKS4 knockout induces EMT-like changes in HCT116 cells [56]. During our experiments, TEER measurements indicated weaker barrier formation in TKS4-KO cells compared with wild-type cells (Figure S1). Although monolayers eventually formed, side-selective labeling was less effective, likely because incomplete junction formation allowed biotinylation reagents to access both membrane domains. Therefore, our analysis focused primarily on changes in CSP abundance rather than polarization.

Several important changes were observed. For example, MMP15 and MMP14 levels decreased on the cell surface in TKS4-KO cells. These matrix metalloproteinases are critical regulators of extracellular matrix degradation and tumor invasion [98,99]. Their reduced surface abundance is consistent with previous observations that TKS4 deficiency disrupts podosome formation and ECM degradation [100].

Similarly, the abundance of ADAM family metalloproteases (ADAM9, ADAM10, ADAM15) was reduced in TKS4-KO cells, while TIMP1, an inhibitor of metalloproteinases, was slightly increased. Other proteins showed increased abundance in knockout cells, including CD59, tissue factor (F3), and uPAR, all of which have been linked to CRC progression. Conversely, several integrins and adhesion proteins—including ITGA3, ITGB1, ITGB5, SLC3A2, and SDC4—were more abundant in wild-type cells, suggesting that TKS4 may regulate their localization or stability at the plasma membrane.

These findings demonstrate that the presented method can detect subtle and large-scale changes in cell surface proteomes resulting from targeted gene disruption. Taken together, our results demonstrate that side-selective biotinylation combined with peptide-level enrichment and LC-MS/MS analysis provides a robust and sensitive strategy for characterizing CRC cell surface proteomes, including protein polarization patterns, glycosylation sites, and gene-dependent alterations.

## 5. Conclusions

To the best of our knowledge, the methods presented here represent the first attempt to achieve both qualitative and quantitative characterization of cell surface proteins (CSPs) in the apical and basolateral membrane domains of different CRC cell lines, including HT29, HCT116, and the gene-edited HCT116-TKS4-KO cells. This approach is based on primary amino group-specific, domain-selective biotinylation, followed by enrichment of labeled peptides or proteins and nanoHPLC–MS/MS analysis.

Identification of surface-accessible regions and domain-specific CSPs in polarized CRC cells provides a framework for improving our understanding of membrane protein organization and polarity-associated differences in CRC models. The datasets generated in this study constitute a valuable resource for future investigations of CRC cell surface architecture and for subsequent studies aiming to evaluate the biological or translational relevance of identified surface-exposed protein regions.

## Figures and Tables

**Table 1:** Labeled proteins detected from HCT116 and HT29 cells using the peptide- and protein-level enrichment strategy. TM means transmembrane.

	HCT116 Peptide Enrichment	HT29 Peptide Enrichment	HCT116 Protein Enrichment	HT29 Protein Enrichment
Labeled proteins	831	1371	274	334
Labeled TM proteins	295	383	134	138

**Table 2:** Comparison of apically labeled proteins identified by us with apically annotated proteins in the Compartments database. Numbers indicate the amount of total apically shifted proteins/labeled apically shifted proteins (apically shifted means that the percentage of the protein is at least 60% apical based on average intensity values in our samples).

	HCT116 Peptide Enrichment	HT29 Peptide Enrichment	HCT116 Protein Enrichment	HT29 Protein Enrichment
Common with Compartments (only apical genes)	65/24	77/20	143/42	159/49
Exclusively showed in our samples	50/20	47/20	113/27	112/32
Total	115/44	124/40	256/69	271/81

**Table 3:** Proteins upregulated and downregulated in HCT116 cells in Ludvigsen et al.’s work. The number indicates how many times were detected for the given gene in our 12 parallel experiments per sample type. The “log intensity” means the log2 value of the summed intensity of the protein in the cell type-specific samples.

Upregulated and Downregulated Genes in Ludvigsen et al. [66]		HCT116 Peptide Enrichment	HCT116 Protein Enrichment	HCT116 log2 Intensity	HT29 Peptide Enrichment	HT29 Protein Enrichment	HT29 log2 Intensity
Upregulated genes	ANXA6	3	11	28.34	5	0	23.55
	CD109	12	12	33.76	3	3	23.82
	COL6A1	0	1	21.10	0	0	-
	CPNE7	9	6	27.31	0	0	-
	DOCK5	12	4	24.89	11	0	24.85
	APOBEC3C	0	1	19.48	0	0	-
	EPHB2	12	12	30.13	9	12	28.15
	PDLIM2	0	0	-	0	2	19.35
	PTPRG	12	12	30.53	1	0	20.18
	SLC2A3	12	12	31.56	0	0	-
	OXCT1	5	3	23.48	0	0	-
	TRIM72	11	9	25.49	0	0	-
	TNFRSF6B	10	6	29.62	0	0	-
	VSNL1	0	10	25.08	0	0	-
Downregulated genes	AKR1B10	0	0	-	8	2	24.28
	AKR1C3	0	0	-	5	4	25.09
	SERPINA1	0	2	23.33	0	0	-
	ANXA10	0	0	-	4	1	22.81
	ANO10	0	2	20.67	0	0	-
	ABCG2	0	3	28.47	1	10	28.04
	TESC	0	0	-	6	10	25.97
	TP53	0	0	-	0	1	19.68
	TMEM63A	1	0	19.73	0	7	23.35
	FAAH	0	0	-	3	0	21.73
	LGALS4	0	0	-	12	12	31.65
	GMDS	0	0	-	12	11	26.69
	GOLIM4	0	0	-	0	2	20.15
	HGD	0	0	-	7	0	24.60
	FCGRT	0	0	-	3	0	22.75
	NAALADL2	0	0	-	4	6	24.31
	RHBDF1	1	0	19.84	11	0	26.54
	LAMA3	12	2	26.44	12	3	28.35
	LY75	0	0	-	12	12	32.65
	MUC13	0	2	23.08	11	12	30.62
	NF1	0	0	-	12	6	25.87
	PLXNA2	0	0	-	12	11	27.92
	PROM2	2	2	21.10	12	12	28.34
	PSMB8	0	0	-	0	12	27.54
	PTPRH	0	0	-	12	12	29.06
	ARHGAP23	0	8	27.23	0	8	27.48
	ITFG1	0	9	26.51	0	11	27.20
	TFPI	9	2	23.97	11	8	25.32
	TMEM62	0	0	-	9	7	25.03
	VIL1	0	0	-	12	12	29.58
	VSIG2	0	0	-	6	12	30.60
	ATP6V1B1	0	0	-	5	1	23.58

**Table 4:** The top 20 genes, which were previously enriched by MGL in [67]; we also characterized each of them using our method. They are sorted by detected intensities (from highest to lowest) the topological correctness of their labeling sites and their distributions in the apico-basolateral membrane domains (indicated by apical percentages for both cell lines and both enrichment strategies). In the column of TM protein, whether the given protein TM (yes) or not (no) was determined by CCTOP TmFilter [68]. Topology OK or notOK columns indicate = how many labeled positions came from the extracellular protein region and how many from the intracellular protein region, respectively (these annotations were assigned based on UniTmp database [63]). The bold apical percentages indicate significant apical or basolateral shifts.

Protein ID	Gene	Protein Name	TM Protein	Topology OK	Topology notOK	Apical % HCT116 Peptide Enrichment	Apical % HT29 Peptide Enrichment	Apical % HCT116 Protein Enrichment	Apical % HT29 Protein Enrichment
P05556	ITGB1	Integrin beta-1	yes	146	0	50.9	52.1	50.3	53.6
P26006	ITGA3	Integrin alpha-3	yes	56	1	53.0	50.0	46.4	51.4
F5GZS6	SLC3A2	Solute carrier family 3 member 2	yes	41	4	55.4	53.3	54.3	55.2
Q9P2B2	PTGFRN	Prostaglandin F2 receptor negative regulator	yes	32	0	54.4	45.8	48.7	49.6
P05023	ATP1A1	Sodium/potassium-transporting ATPase subunit alpha-1	yes	0	9	49.6	49.7	52.1	53.3
P10586	PTPRF	Receptor-type tyrosine-protein phosphatase F	yes	34	1	50.7	50.3	51.0	49.9
Q13308	PTK7	Inactive tyrosine-protein kinase 7	yes	29	0	**56.4**	54.4	49.6	**57.6**
P11279	LAMP1	Lysosome-associated membrane glycoprotein 1	yes	6	0	43.6	43.6	54.5	51.4
P02786	TFRC	Transferrin receptor protein 1	yes	33	0	**62.4**	54.2	59.1	55.3
O75054	IGSF3	Immunoglobulin superfamily member 3	yes	36	0	51.8	45.6	51.7	49.2
P15529	CD46	Membrane cofactor protein	yes	30	0	48.5	53.0	36.0	53.8
P08581	MET	Hepatocyte growth factor receptor	yes	28	0	55.3	**56.4**	49.7	**60.4**
O75369	FLNB	Filamin-B	no	0	47	25.1	**38.4**	48.0	44.7
Q92896	GLG1	Golgi apparatus protein 1	yes	9	0	50.8	54.8	54.6	50.3
E9PGC5	PTPRK	protein-tyrosine-phosphatase	yes	17	0	56.1	52.3	40.2	50.2
P51572	BCAP31	B-cell receptor-associated protein 31	yes	2	9	43.9	56.4	53.7	**61.3**
Q92673	SORL1	Sortilin-related receptor	yes	0	0	43.1	41.2	49.1	**67.3**
Q8NBJ4	GOLM1	Golgi membrane protein 1	yes	2	0	39.4	NaN	58.4	51.3
Q6ZRP7	QSOX2	Sulfhydryl oxidase 2	yes	1	0	NaN	NaN	NaN	NaN
P20023	CR2	Complement receptor type 2	yes	1	0	NaN	NaN	NaN	NaN

**Table 5:** The number of detected *N*-glycoproteins, transmembrane *N*-glycoproteins and their topological correctness. Numbers in the brackets are the unique/different *N*-glycosylation sites in the sample types. TopOK sites %: Percentage of topologically correct extracellular *N*-glyco sites of transmembrane (TM) proteins compared to total *N*-glyco sites of TMPs.

	HCT116 Peptide Enrichment	HT29 Peptide Enrichment	HCT116 Protein Enrichment	HT29 Protein Enrichment
*N*-glycoproteins	232	257	198	212
*N*-glycosylation sites	822 (368)	742 (386)	398 (295)	378 (295)
TM *N*-glycoproteins	146	139	87	69
*N*-glyco sites of TMPs	622 (257)	497 (241)	224 (157)	149 (111)
TM topology OK sites	613 (251)	489 (235)	188 (127)	127 (94)
TM topology notOK sites	9 (6)	8 (6)	36 (30)	22 (17)
TopOK sites %	99	98	84	85

## Data Availability

Database search parameters and raw result files are available at https://doi.org/10.5281/zenodo.19629045.

## Supplementary Materials

The following supporting information can be downloaded at: https://www.mdpi.com/article/10.3390/biom16060865/s1. Supplementary documents contain Materials and methods section and Results sections. Moreover, we have uploaded three separate Supplementary tables as follows: Supplementary Table S1. List of the identified and quantified peptides and proteins from CRC cell lines. See Supplementary_Table_S1.xlsx; Supplementary Table S2. List of the identified and quantified peptides and proteins from wild-type and TKS4-KO CRC cell lines. See Supplementary_Table_S2.xlsx; Supplementary Table S3. Glycosylation sites containing peptides from CRC cell lines. See Supplementary_Table_S3.xlsx.

## Abbreviations

The following abbreviations are used in this manuscript:

CCTOP	Consensus Constrained TOPology prediction
CIRFESS	Compiled Interactive Resource for Extracellular and Surface Studies
CRC	Colorectal cancer
CSP(s)	Cell surface protein(s)
EMT	Epithelial-to-mesenchymal transition
LC-MS/MS	Liquid chromatography–tandem mass spectrometry
LFQ	Label-free quantification
MAPs	Membrane-anchoring proteins
MDCKII	Madin-Darby canine kidney cell line
MGL	Macrophage galactose-type lectin
MMPs	Monotopic membrane proteins
PX	Phox homology domain
SH3	Src homology 3 domain
TEER	Transepithelial electrical resistance
TMP(s)	Transmembrane protein(s)
UniTmp	UNIfied database of TransMembrane Proteins
