# Supplementary material for: Decoding the Apical–Basal Surfaceome of Colon Epithelial Cells via Side-Selective Biotinylation
Source: Biomolecules. 2026 Jun 12;16(6):865. doi: 10.3390/biom16060865 (PMC13296770; doi:10.3390/biom16060865)
Supplement: Supplementary file 1 [file biomolecules-16-00865-s001.zip › Supplementary_information.pdf]

Supplementary information for

Decoding the Apical-Basal Surfaceome of Colon Epithelial Cells via Side-Selective Biotinylation

Katalin Kuffa [1,2,+], Tamás Langó [2,+], András Czirók [3], Júlia Tárnoki-Zách [3], Szilvia Bősze [4,5], Loretta László [2], Virág Vas [2], Zoltán Szabó [2,6,*], and Gábor E. Tusnády [2,7,*]

[1] Doctoral School of Biology, Institute of Biology, ELTE Eötvös Loránd University, Pázmány P. stny. 1/C, H-1117 Budapest, Hungary

[2] Protein Bioinformatics Research Group, Institute of Molecular Life Sciences, Research Centre for Natural Sciences, HUN-REN, Magyar Tudósok körútja 2, H-1117 Budapest, Hungary

[3] Department of Biological Physics, Eötvös Loránd University, Pázmány P. stny. 1/A, H-1117 Budapest, Hungary

[4] HUN-REN-ELTE Research Group of Peptide Chemistry, Hungarian Research Network, Eötvös Loránd University, Pázmány P. stny. 1/A, H-1117 Budapest, Hungary

[5] Department of Genetics, Cell- and Immunobiology, Faculty of Science, Semmelweis University, Nagyvárad tér 4, H-1089 Budapest, Hungary

[6] Department of Medical Chemistry, Albert Szent-Györgyi Medical School, University of Szeged, H-6725 Szeged, Hungary

[7] Department of Bioinformatics, Semmelweis University, Tűzoltó u. 7, H-1094 Budapest, Hungary

[+] These authors have contributed equally to the work.

*Correspondence: tusnady.gabor@ttk.hu, szabo.zoltan@med.u-szeged.hu

Supplementary Materials and Methods

Dot blot method for biotinylation measurements

We have developed a Dot blot analysis to compare the biotinylation levels of various membrane preparations (such as biotinylated apical or basolateral membranes from polarized CRC cells, biotinylation process is detailed under the “Side-selective Sulfo-NHS-SS-biotin labeling process” subheading) originated from HL60, HCT-116 wt, HCT-116 TKS4-KO and HT-29 cells. Based on protein measurement (Lowry et al. method was applied, [58]), serial dilutions were prepared (at least two/three dilutions per sample) between 0.2 µg to 0.05 µg protein content and stored them on ice. First, the polyvinylidene difluoride (PVDF) membrane (0.22 or

0.45 µm pore size) was activated with methanol for 5 min in a plastic container under constant shaking. Then the activation buffer was discarded and the membrane was incubated with TBS-T buffer (25 mM Tris-HCl, pH=7.4, 2.7 mM KCl, 137 mM NaCl, 0.05% (V/V) Tween-20) for 5 min again (while shaking gently to avoid the drying out of the membrane). The PVDF membrane was inserted into the Bio-rad 96-well Bio-Dot microfiltration device (under the PVDF membrane we used a TBS-T wetted filter paper) and the diluted samples were loaded into the wells. The biotinylated peptides/proteins were directly immobilized onto the PVDF membrane using a vacuum pump conducted Bio-Dot microfiltration apparatus. The vacuum pump was turned off after two minutes and the PVDF membrane was washed with TBS-T solution several times. Finally, the membrane was blocked with 2% (m/V) bovine serum albumin (BSA) in TBS-T buffer for 60 min at room temperature, then blocking buffer was discarded and treated by horseradish peroxidase (HRP) conjugated streptavidin (Thermo Fisher Scientific) for 60 min in dark (HRP conjugated streptavidin was diluted at a ratio of 1:50000 in the blocking buffer). Then the PVDF was washed three times with TBS-T, followed by 10 minutes of incubation for each wash step. The biotinylation efficiency was visualized by HRP-specific substrate, the enhanced chemiluminescence reagent (Merck Millipore Ltd, luminol and peroxide solutions were mixed in 1:1 ratio immediately before use), and the images were captured by a ChemiDoc XRS+ Imaging system (Bio-Rad). The intensity of the spots was determined by Image Lab 6.0 software (Supplementary Figure S2A).

In order to bind all biotinylated peptides or proteins on the high capacity NeutrAvidin agarose resin, samples were taken from the before column fractions and the after column fractions (flow-through fractions), and were also monitored by Dot Blots similarly as described above (Supplementary Figure S2B).

Western blot analysis

HCT116 WT and TKS4-KO cells were isolated from T25 flasks in ice-cold Harvest buffer (30 mM Tris (pH 7.5), with 100 mM NaCl, 1 mM EGTA, 1% Triton X-100, 10 mM NaF, 2 mM 4-nitrophenyl phosphate, 10 mM benzamidine, 25 µg/mL trypsin inhibitor, 25 µg/mL Pepstatin A, and 25 µg/mL aprotinin and completed on the day of use with 1 mM Na_3VO_4, 1 mM phenylmethylsulphonyl fluoride) using a cell scraper. The cell lysates were centrifuged at 20,000 g for 10 minutes at 4 °C. The supernatants were transferred into new Eppendorf tubes and the protein content of the isolates was determined by the method of Lowry et al. 30-30 µg

protein containing WT and TKS4-KO cell lysates were diluted in 4x Laemmli sample buffer and boiled for 5 minutes at 95 °C in a dry-block thermostat, then the samples and the protein ladder (GRiSP, cat. no. GLP01.0500) were loaded into the gel wells. Electrophoresis was performed for 60 min at 140 V in electrophoresis buffer (25 mM Tris, 192 mM glycine and 0.1% (w/v) SDS) using a PowerPac Universal power supply with a Mini-Protean tetra vertical electrophoresis cell.

Proteins were transferred to PVDF membrane from the gel using the transfer buffer (25 mM Tris, 192 mM glycine and 15% (v/v) methanol) at 70 V for 3 hours. PVDF membranes were blocked with 5% (w/v) Bovine Serum Albumin (BSA, Merck Sigma-Aldrich, cat. no. A7906) in TBS-T (25 mM Tris-HCl, pH 7.4, 2.7 mM KCl, 137 mM NaCl, 0.1% (v/v) Tween-20) and incubated for 60 minutes to reduce nonspecific antibody binding. Subsequently, the appropriate PVDF regions were incubated overnight at 4 °C with the primary antibodies (such as polyclonal rabbit anti-TKS4 antibody from Lányi et al. [85], dilution 1:1000; HPA036471 polyclonal rabbit anti-TKS4 antibody, Sigma-Aldrich, dilution 1:1000 and DM1A monoclonal mouse anti-tubulin antibody, Sigma-Aldrich, dilution 1:1000 in 5% BSA in TBS-T buffer). Next day, after three washing steps, the membranes were incubated for 60 minutes with horseradish peroxidase-conjugated secondary antibodies (anti-rabbit and anti-mouse antibodies, Thermo Fisher Scientific, dilution: 1:2500 and 1:1000, respectively) and then washed three times for 10 min each. The bands of the proteins are visualized by ChemiDoc XRS+ Imaging system (Bio-Rad) via enhanced chemiluminescence (ECL) detection reagents (Merck Sigma-Aldrich, see Figure S5A).

ICC and confocal microscopy method

HCT116 WT and TKS4-KO cells were seeded into a 12-well removable ibidi chamber (ibidi, cat. no. 81201) at a density of 5,000 cells per well. Cells were fixed with 4% paraformaldehyde (PFA) for 15 minutes at room temperature (RT), followed by washing with Dulbecco’s phosphate-buffered saline (DPBS; Merck Sigma-Aldrich, cat. no. D8537). Permeabilization was performed using 0.1% Triton X-100 in sterile DPBS for 10 minutes at RT.

To reduce nonspecific binding, cells were blocked with 5% bovine serum albumin (BSA) in DPBS for 1 hour at RT. The samples were then incubated overnight at 4 °C with TKS44 primary antibodies (polyclonal rabbit antibody from Lányi et al.[85], dilution 1:2000, or HPA036471, Sigma-Aldrich, dilution 1:400). After multiple DPBS washes, cells were incubated with Alexa

Fluor 488-conjugated anti-rabbit secondary antibody (cat. no. A-11008; Thermo Fisher Scientific) for 1 hour at RT.

Cell nuclei were stained with DAPI (Thermo Fisher Scientific, cat. no. 62248) for 10 minutes at RT. Following a final wash with DPBS (3 minutes, RT), the silicone chamber was removed, and samples were mounted using FluorSave mounting medium (Merck Millipore, cat. no. 345789) with a coverslip. Slide edges were sealed with nail polish. Imaging was performed using a Zeiss LSM 710 confocal microscope equipped with a 40× objective (see Figure S5B).

Supplementary Results

Figure S1. Trans-Epithelial Electrical Resistances (TEER) for the appropriate cell types depending on the days of growth.

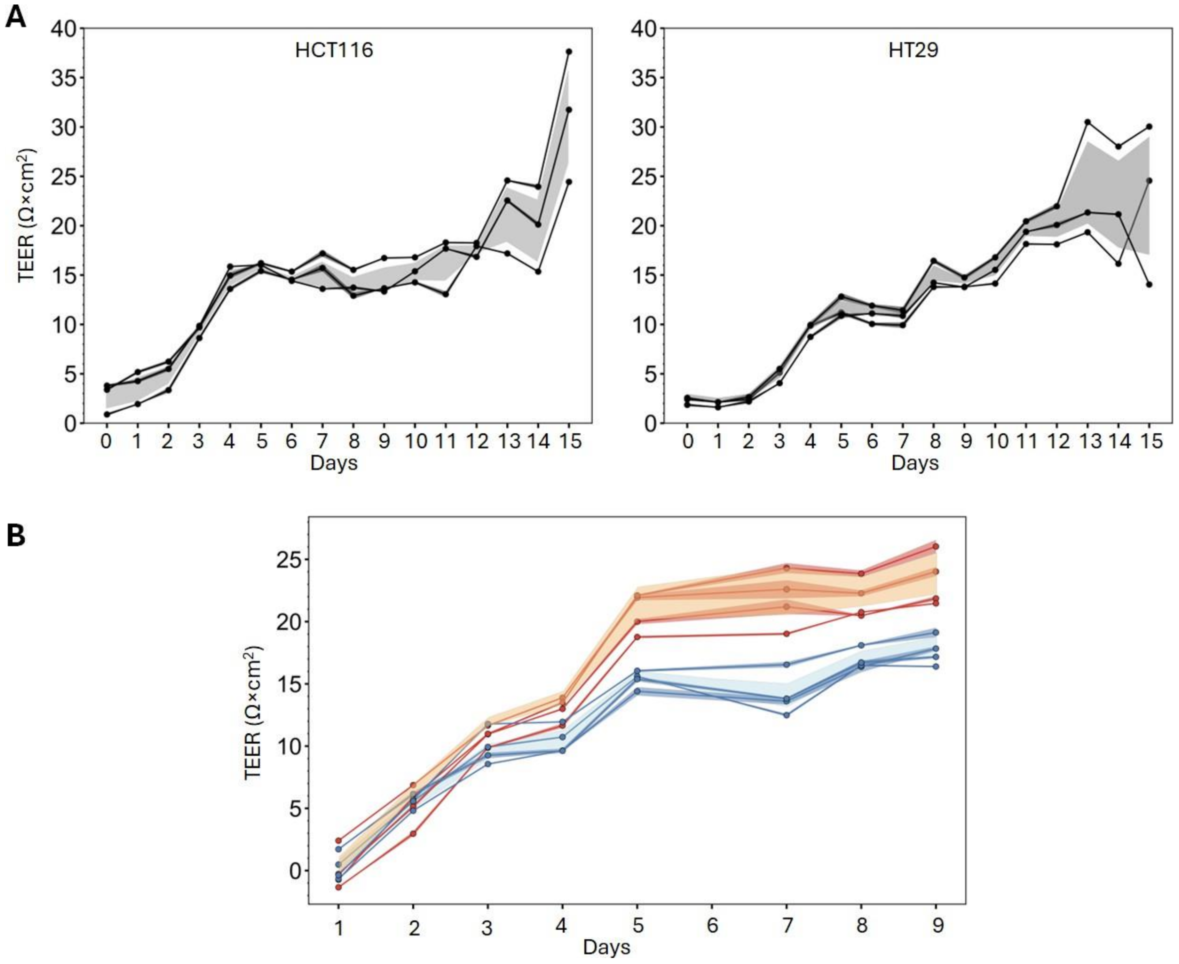

Figure S1. Cells were seeded on transwell inserts, and TEER values were monitored daily (as described in detail in Kuffa et al., 2025 [45]) to identify the time point at which resistance values stabilized. The emergence of these plateaus is consistent with the formation of a continuous cell layer that measurably limits ionic currents across the insert. **(A)** In the upper left and upper right panels show the time course of TEER values for CRC cells over 15 days (HCT116 and HT29, respectively). **(B)** HCT116 wild-type and HCT116 TKS4 KO cells were analyzed by TEER measurements in 4-4 transwell inserts for nine days (indicated by red and blue lines, respectively). TEER values from parallel cultures are shown. Shaded areas indicate interquartile ranges for all cultures of the specific cell.

Figure S2. Dot blot analysis to determine the biotinylation efficiency of apical or basolateral membrane domains, as well as to monitor the binding of biotinylated components on affinity column.

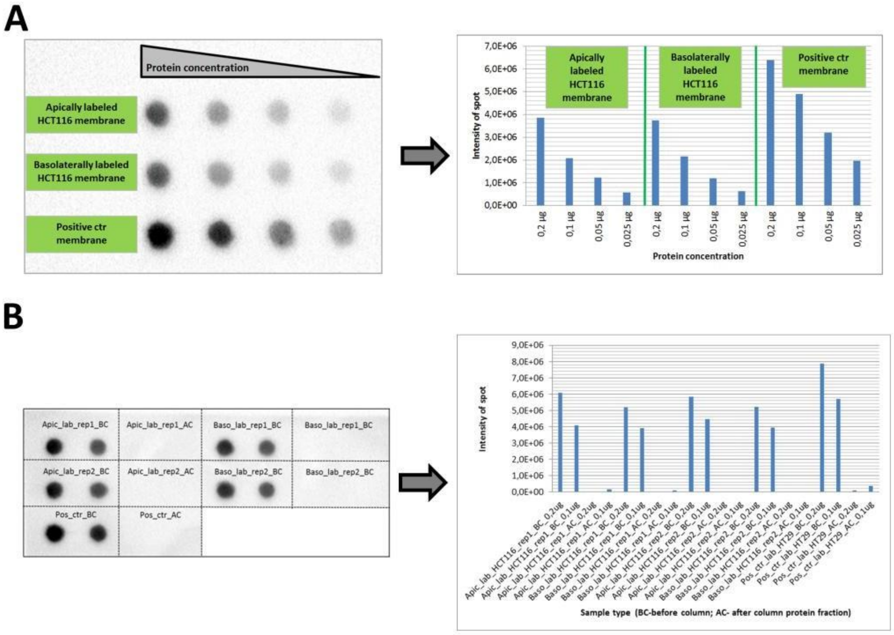

Figure S2. The horseradish peroxidase (HRP)-catalyzed chemiluminescent intensity values of the dot blot measurements to detect the biotinylation efficacy in different Sulfo-NHS-SS-biotin labeled membrane/protein fractions (such as membrane preparations and solubilized labeled protein fractions before and after avidin column; A, B respectively). (A) Comparative analysis of side-selectively labeled membranes. HCT116 cells were cultured in transwell inserts and the cell layers were labeled from the apical or from the basolateral chamber, after the reactions were stopped and the side-selectively labeled membrane preparations were isolated. Based on protein measurements two-fold dilution series were prepared from both apical and basolateral biotinylated membranes (with a protein content between from 0.2 µg to 0.025 µg, the positive control was a biotinylated HL60 membrane preparation and it was diluted in a similar range). These dilution series were spotted directly onto a PVDF membrane and the biotinylation efficacy was visualized by HRP-conjugated streptavidin. The raw result of the blot is shown in the left side and the integrated spot intensities are shown in the right side. (B) Monitoring the

binding of the biotinylated components on neutravidin agarose column. Apically or basolaterally labeled HCT116 membrane preparations were solubilized (2-2 biological replicates are/were analyzed, that are indicated by rep1 and rep2) and their biotin contents are immobilized on neutravidin agarose column. 0.2 µg and 0.1 µg protein containing before column fractions (BC) and after column fractions (AC) were blotted directly onto a PVDF membrane, and visualized as mentioned above (raw data and integrated intensities are shown left and right, respectively; positive control was a labeled HT29 membrane preparation). Images of dot blots were captured by a Bio-Rad ChemiDoc XRS+ Imaging system. The intensities of the spots were determined by Image Lab 6.0 software in all dot blot experiments.

Biological and technical replicates and overall protein reproducibility, labeling efficiency and accuracy

In this study, we investigated the cell surface proteomes of two CRC cell lines (HCT116 and HT29), as well as a gene-edited derivative of HCT116 (HCT116-TKS4-KO). For the wild-type (WT) CRC cell lines, three biological replicates were prepared from both the apical and basolateral labeled surfaces. Each sample was analyzed in two technical replicates using nanoHPLC-MS/MS. Surface-labeled proteins were enriched both prior to and following trypsin digestion, enabling enrichment at both the protein and peptide levels. In total, 3 (biological replicates) × 2 (cell lines) × 2 (cell surfaces) × 2 (technical replicates) × 2 (enrichment strategies) = 48 mass spectrometry (MS) runs were performed for the CRC cell lines.

For the HCT116 -TKS4-KO cells, we have also grown them in transwell inserts. Both the knockout (KO) and their isogenic WT counterparts (i.e., the parental line from which the KO was derived) were subjected to surface protein labeling. Enrichment was again performed at both the protein and peptide levels, with two technical replicates per condition. In total, 2 (apical, basolateral cell surface biotinylation) × 2 (cell types: WT and KO) × 2 (enrichment strategies) × 2 (biological replicates) = 16 MS runs were accomplished.

We observed strong reproducibility between biological and technical replicates based on Supplementary Table 1-2 (peptide enrichment: protein overlap range: 75%-82%; Pearson’s R: 0.96–0.98; protein enrichment: protein overlap range: 44%–78%; Pearson’s R: 0.87%–0.98%; for the comparison of biological replicates, only those peptides detected in both technical replicates were considered).

To ensure that the labeling procedure specifically targeted CSPs, we developed a bioinformatics-based quality control protocol, building upon our previous work. The core of this method involves assessing the ratio of peptides labeled on the correct (extracellular) side versus all labeled peptides. The topologies of the transmembrane proteins were predicted using CCTOP, a topology prediction tool developed by our group.

In all cases, peptides labeled on the incorrect intracellular side were distributed toward lower peptide counts and lower intensities compared to those labeled on the correct extracellular side (based on Supplementary Table 1-2). For the HCT116 cell line, the proportion of incorrectly labeled peptides remained below 6% across all biological replicates. However, in the HT29 cell line, this proportion occasionally reached up to 10%, suggesting a higher sensitivity of this cell line to the labeling procedure. This may reflect increased cell lysis during labeling, allowing the reagent to access the intracellular sides of membrane proteins. In subsequent data analysis, we only considered those proteins for which labeled peptides were confidently identified on the correct, extracellular side.

Mass spectrometry analysis details

All LC-MS measurements were performed on a Waters ACQUITY UPLC M-Class LC system (Waters, Milford, MA, USA) coupled with an Orbitrap Exploris 240 mass spectrometer (Thermo Fisher Scientific, Waltham, MA, USA). A symmetry C18 (100 Å, 5 µm, 180 µm × 20 mm) trap column was used for trapping and desalting the samples. The chromatographic separation of peptides was accomplished on an ACQUITY UPLC M-Class Peptide BEH C18 analytical column (130 Å, 1.7 µm, 75 µm × 250 mm) at 45 °C by gradient elution. Water (solvent A) and acetonitrile (solvent B), both containing 0.1% formic acid, were used as mobile phases at a flow rate of 200 nL/min. The sample temperature was maintained at 5 °C. The mass spectrometer was operated using the equipped Nanospray Flex Ion Source. Measurements were collected using a data dependent analysis (DDA) method with an MS1 scan between 360 and 2200 Th using 60,000 resolution, while ddMS2 scans with isolation windows of 2 Th were collected at 30,000 resolution, keeping a 3 s cycle time. Data acquisition was performed using XcaliburTM 4.6 (Thermo Fisher Scientific, Waltham, MA, USA). Raw LC-MS data files were processed using Fragpipe v22.0. A human reference proteome including common contaminants (83,676 proteins) was used for protein identification. Trypsin as digestion enzyme assuming two missed cleavage, and Met oxidation, 3-(carbamidomethylthio)propanoyl on Lys and

protein N-terminal (effect of biotin labelling), pyro Glu and carbamidomethyl on Lys an peptide N-terminal as variable and on Cys as fixed modifications were considered. Protein quantification was performed within Fragpipe using IonQuant with default settings and enabling MBRs. Statistical analysis and visualization of proteomics data was performed in Perseus 1.6.15145 and Instantclue v0.12.2146. Protein and peptide intensity data were log2 transformed and median normalized before statistical analysis. Differential expression analysis (DEA) was performed in Perseus using the built in Student's t-test using permutation-based false discovery rate (FDR) estimation with a limit of FDR<0.05.

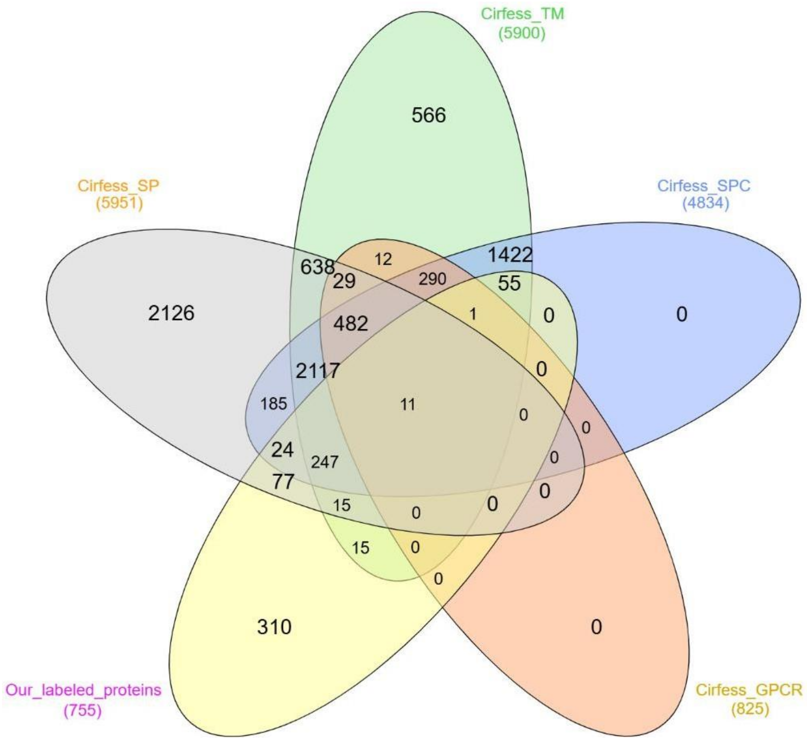

Figure S3. Comparison of protein hits with Cirfess databases containing CSP-specific data. We compared data from the Cirfess database [60] with proteins which were identified with at least one extracellular (topologically correct) labeled position in the present work. (SP: signal peptide, TM: transmembrane, SPC: surface prediction consensus, GPCR: G-protein coupled receptor)

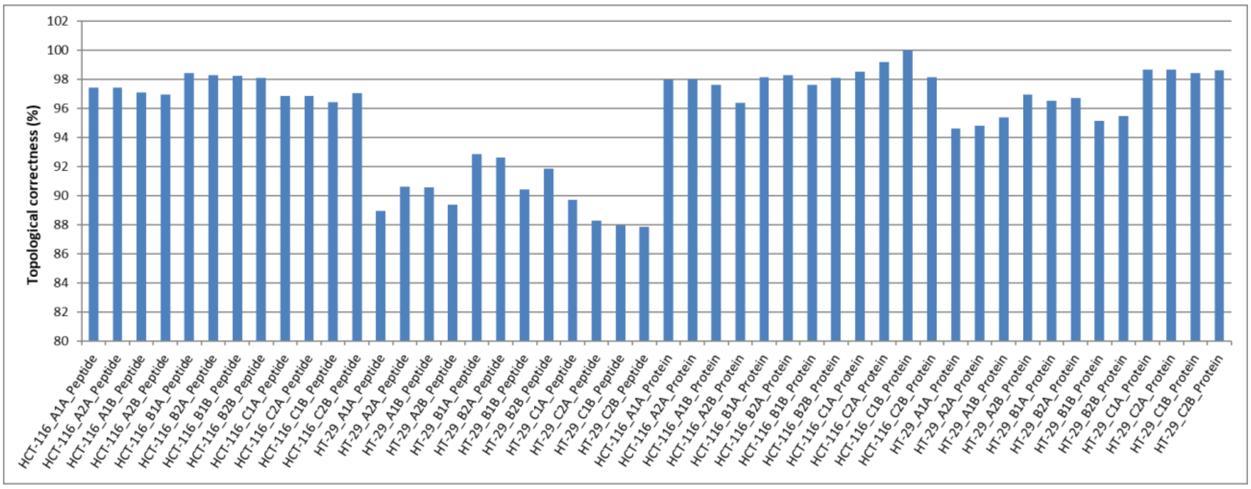

Figure S4. Topological correctness of labeled TM peptides in the HCT116 and HT29 samples. This figure shows the ratio of labeled transmembrane (TM) peptides on the correct (extracellular) side versus all labeled TM peptides. "Peptide" means labeled peptide enrichment, "Protein" means labeled protein enrichment strategy. A1A: A means the first biological replicate, 1 means the first technical replicate, A denotes apically labeled sample.

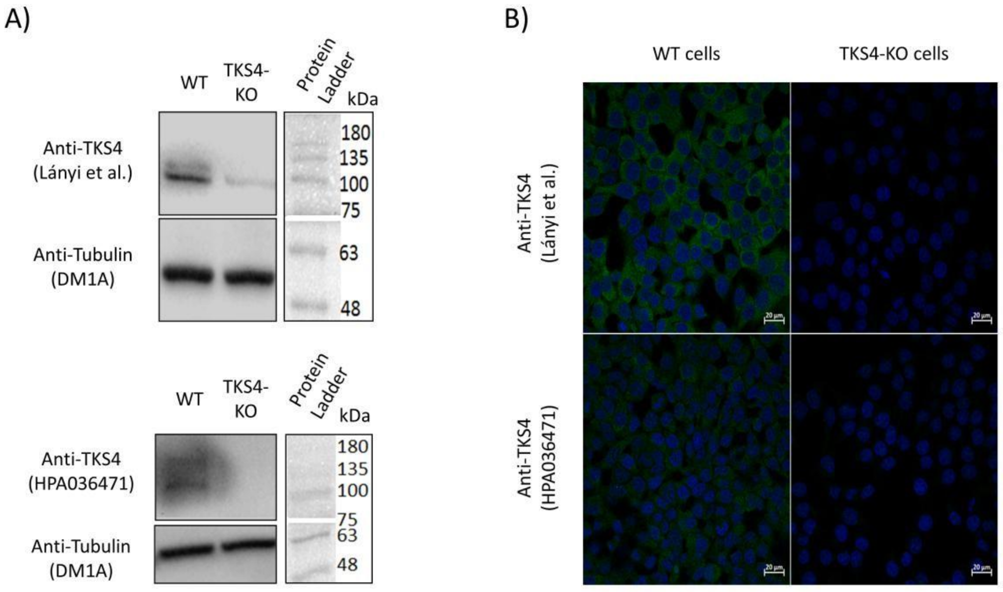

Figure S5. Validation of the absence of TKS4 protein via Western blot and immunocytochemistry. A) The lack of TKS4 protein expression was confirmed by Western blot with two different polyclonal anti-TKS4 antibodies. B) Representative fluorescent images of wild-type (WT) and TKS4-KO HCT116 cells, where nuclei are visualized with DAPI (blue), while TKS4 is shown in green. Scale bar represents 20 µm.

Supplementary Tables

Supplementary Table S1. List of the identified and quantified peptides and proteins from CRC cell lines

see Supplementary_Table_1.xlsx

Supplementary Table S2. List of the identified and quantified peptides and proteins from wild-type and TKS4-KO CRC cell lines

see Supplementary_Table_2.xlsx

Supplementary Table S3. Glycosylation sites containing peptides from CRC cell lines

see Supplementary_Table_3.xlsx
